# Simplexide Induces CD1d-Dependent Cytokine and Chemokine Production from Human Monocytes

**DOI:** 10.1371/journal.pone.0111326

**Published:** 2014-11-12

**Authors:** Stefania Loffredo, Rosaria I. Staiano, Francescopaolo Granata, Valeria Costantino, Francesco Borriello, Annunziata Frattini, Maria Teresa Lepore, Alfonso Mangoni, Gianni Marone, Massimo Triggiani

**Affiliations:** 1 Department of Translational Medical Sciences and Center for Basic and Clinical Immunology Research (CISI), University of Naples Federico II, Naples, Italy; 2 The NeaNAT group - Department of Pharmacy, University of Naples Federico II, Naples, Italy; 3 Division of Allergy and Clinical Immunology, University of Salerno, Salerno, Italy; University of Leuven, Rega Institute, Belgium

## Abstract

Monocytes are major effector cells of innate immunity and recognize several endogenous and exogenous molecules due to the expression of wide spectrum of receptors. Among them, the MHC class I-like molecule CD1d interacts with glycolipids and presents them to iNKT cells, mediating their activation. Simplexide belongs to a novel class of glycolipids isolated from marine sponges and is structurally distinct from other immunologically active glycolipids. In this study we have examined the effects of simplexide on cytokine and chemokine release from human monocytes. Simplexide induces a concentration- and time-dependent release of IL-6, CXCL8, TNF-α and IL-10 and increases the expression of *IL6*, *CXCL8* and *IL10* mRNA. Cytokine and chemokine release induced by simplexide from monocytes is dependent on CD1d since: i) a CD1d antagonist, 1,2-bis (diphenylphosphino) ethane [DPPE]- polyethylene glycolmonomethylether [PEG], specifically blocks simplexide-induced activation of monocytes; ii) CD1d knockdown inhibits monocyte activation by simplexide and iii) simplexide induces cytokine production from CD1d-transfected but not parental C1R cell line Finally, we have shown that simplexide also induces iNKT cell expansion *in vitro*. Our results demonstrate that simplexide, apart from activating iNKT cells, induces the production of cytokines and chemokines from human monocytes by direct interaction with CD1d.

## Introduction

Monocytes are a critical component of the mononuclear phagocyte system and play an important role in conditions as diverse as infections, cardiovascular diseases and cancer [1], [2], [3]. By expressing a wide spectrum of surface receptors, monocytes can recognize several chemically unrelated molecules (e.g. proteins and lipids) [4], [5], [6], [7]. Glycolipids and glycosphingolipids are major components of several microorganisms, and are increasingly recognized as potent activators of immune cells [8], [9]. These molecules can interact with the MHC class I-like molecule CD1d expressed on antigen presenting cells (APC), such as dendritic cells and monocytes [10], [11].

Natural killer T cells with an invariant T cell receptor alpha chain (iNKT) recognize microbial glycolipids bound to and presented by CD1d [12], [13], [14]. α-Galactosylceramide (α-GalCer), a glycolipid extracted from the marine sponge *Agelas mauritiana* [15], stimulates NKT cells to rapidly produce both Th1 (IFN-γ) and Th2 (IL-4) cytokines in a CD1d-dependent manner [16], [17]. Although it is well established that presentation of the CD1d-α-GalCer complex by APC to iNKT cells results in their activation [17], [18], [19], it has been demonstrated that direct cross-linking of CD1d on human monocytes [20], [21] and intestinal epithelial cells induces cytokine production [22], [23].

**Figure 1:**
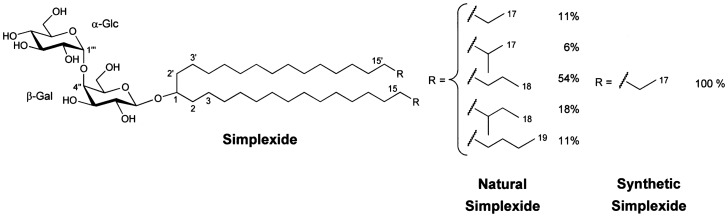
Chemical structure of simplexide. Simplexide is a glycolipid composed of long-chain secondary alcohols glycosylated by a disaccharide chain containing α-glucose and α-galactose. Natural simplexide is a mixture of homologues, with alkyl chains of different length (R) linked to the central CHOH group. In addition, a significant part of the alkyl chains has a methyl branch in the second-to-last or third-to-last carbon atoms. The percentage of each molecular species detected in natural simplexide is shown on the right. Synthetic simplexide was prepared as a chemically homogeneous compound.

Simplexide is the leading compound of a unique glycolipid class isolated from the sponge *Plakortis simplex* [24], [25]. The lipid component of simplexide is unique among known classes of glycolipids, being a glycosylated long-chain secondary alcohol without further functional groups. The two lipophilic long alkyl chains are linked to a polar sugar head composed of the rare α-glucosyl-(1→4)-β-galactosyl disaccharide residue (Fig. 1). At least five different alkyl chain types are observed, and since there are two alkyl chains for molecule, up to 25 different molecular species may be present in natural simplexide. Although there is some early evidence that simplexide can modulate murine T cells [26], the effects of this molecule on human immune cells are unknown.

In this study we demonstrate that simplexide activates human monocytes and induces the production of cytokines and chemokines in a CD1d-dependent manner.

## Materials and Methods

### Reagents

The following were purchased: lipopolysaccharide (LPS; from *Escherichia coli* serotype 026:B6), L-glutamine, ultraglutamine, antibiotic-antimycotic solution (10,000 IU/ml penicillin, 10 mg/ml streptomycin, and 25 µg/ml amphotericin B), MEM non essential amino acids solutions, Triton X-100, polymyxin B sulfate, Histopaque-1077, bovin serum albumin (BSA) and phorbol-12-myristate-13-acetate (PMA) (Sigma-Aldrich, St. Louis, MO); X-VIVO 15, Pen-Strep, and human AB serum (Lonza, Swiss); RPMI and fetal calf serum (FCS) (MP Biomedicals Europe, Illkirch, France). Human recombinant interleukin (IL)-2 (Peprotech, Italy). Mouse monoclonal IgG1 anti-human CD1d (Clone NOR3.2/13.17), goat polyclonal anti-human GAPDH, HRP-conjugated goat anti-mouse Ab, and HRP-conjugated rabbit anti-goat Ab were from Santa Cruz Biotechnology (Santa Cruz, CA). PE-anti-TCR Vα24 and PerCP-anti-CD3 were from Miltenyi Biotec (Bologna, Italy). FITC-anti-CD14, APC- and PE-anti-CD1d (Clone CD1d42) were from Becton Dickinson (San Jose, CA). 1,2-Bis(diphenylphosphino)ethane[DPPE]-polyethylene glycolmonomethylether[PEG] was from Avanti Polar Lipids (Alabaster, AL). Target-specific primers for *IL6*, *TNFα*, *IL10*, *CXCL8*, and *GAPDH* were designed using the Beacon Designer 3.0 (Biorad Laboratories, Milan, Italy) and produced and purified by Custom Primers (Life Technologies, Milan, Italy). All other reagents were from Carlo Erba (Milan, Italy).

### Isolation and characterization of simplexide

Simplexide was isolated from Plakortis simplex at the Department of Pharmacy, University of Naples Federico II [26], [27]. The structure and purity of the isolated glycolipids was confirmed by Proton Nuclear Magnetic Resonance (^1^H-NMR) and mass spectrometry [26]. As with most glycolipids from sponges, each of the obtained glycolipids was an inseparable mixture of homologues, which are identical in the polar part of the molecule but slightly different in the length and branching of lipophilic chains. The relative amounts of branched and unbranched chains were evaluated by ^1^H-NMR, while their length was determined by mass spectrometry. Both resulted to be very close to those reported in the original paper [24], [27]. Structure reported in Figure 1 shows the relative amounts of homologues of natural simplexide. Stock solutions of glycolipid were prepared and stored in DMSO at a concentration of 3 mM unless otherwise specified and diluted to working concentration in RPMI immediately before the experiment. Synthetic simplexide was prepared from 1-octatecanol, 1-bromoheptane, methyl α-d-glucopyranose, and methyl β-d-galactopyranose (see Figure S1). Final purification of the synthetic compound was achieved using reversed-phase HPLC using an RP-18 column and MeOH as eluent. Natural α-GalCer was isolated from the marine sponge Agelas longissima using the same procedure as for simplexide. Synthetic α-GalCer (KRN7000) [28] was purchased from Cayman Chemical (Michigan, USA).

### Cell isolation and purification

**Figure 2:**
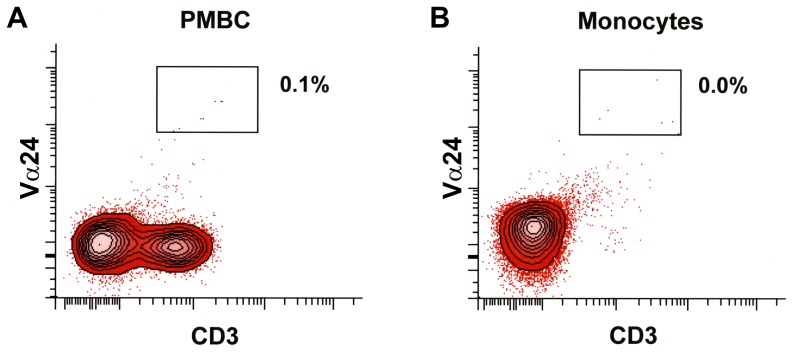
Flow cytometric analyses of iNKT cells among human PBMC and in highly purified monocytes. The percentage of iNKT cells in PBMC (A) and in monocytes (B) was analyzed by flow cytometry with anti-Vα24 and anti-CD3. The cells washed in PBS, were incubated at 4°C for 20 minutes with antibody mixes: PE-conjugated TCR Vα24 and PerCP-conjugated anti- CD3. For each sample 30,000 events were acquired on BD LSRFortessa. All flow cytometric analyses were performed using a BD FACSDiva software.

The study protocol involving the use of human blood cells was approved by the Ethical Committee of the University of Naples Federico II, and written informed consent was obtained from blood donors in according to the principles expressed in the Declaration of Helsinki. Monocytes were purified from buffy coats of healthy donors (HCV^-^, HBsAg^-^, HIV^-^) obtained from the Leukapheresis Unit. Peripheral blood mononuclear cells (PBMC) were obtained by centrifugation over Histopaque-1077. Monocytes were further purified by positive immunomagnetic selection using CD14 MicroBeads (Miltenyi Biotec, Bologna, Italy). This procedure yields a population of CD14^+^ monocytes with a purity greater than 99% as assessed by flow cytometry. Contaminating cells were predominantly CD3^+^ T cells. The presence of iNTK cells before and after immunomagnetic selection was assessed by flow cytometry using PE-anti-TCR Vα24 and PerCP-anti-CD3 antibodies. iNTK cells (identified as CD3^+^ Vα24^+^ cells) represented 0.1% of PBMC, whereas these cells were undetectable in monocyte preparations (Fig. 2).

The human C1R and stable CD1d-transfected C1R cell lines [29] were kindly donated by Prof. Vincenzo Cerundolo (Weatherall Institute of Molecular Medicine University of Oxford John Radcliffe Hospital). The cells were maintained in culture in RPMI supplemented with 10% FCS, 2 mmol/L L-glutamine, Pen-Strep and non essential amino acid. The expression of CD1d was assessed by flow cytometry using PE-conjugated anti-CD1d (Clone CD1d42; BD Pharmingen).

### Flow cytometry

Cells were washed in staining buffer (phosphate buffered saline [PBS], 10% human AB serum, 0.05% NaN_3_) and incubated at 4°C for 20 minutes with the following antibodies: PE-anti-TCR Vα24 (dilution 1∶20), PerCP-anti-CD3 (dilution 1∶20) and FITC-anti-CD14 (dilution 1∶20). Then, cells were washed in washing buffer (PBS, 0.2% BSA, 0.05% NaN_3_) and fixed in 2% paraformaldehyde before acquisition on a BD LSRFortessa. For each sample 30,000 events were acquired and analyzed with the BD FACSDIVA software system (Becton Dickinson).

### Cell incubations

Monocytes were incubated (37°C, 2 to 24 h) in X-VIVO supplemented with 2 mM L-glutamine and various concentrations of simplexide (0.3–10 µM) or LPS (100 ng/ml). Simplexide preparations were routinely checked for LPS contamination (Limulus amebocyte Test, MP Biomedicals) and discarded if the LPS concentration was above the detection limit of the assay (0.125 EU/ml). In selected experiments simplexide (10 µM) and LPS (100 ng/ml) were preincubated (37°C, 30 min) with polymyxin B (50 µg/ml) before addition to the cells. Since synthetic simplexide was remarkably less soluble in DMSO than natural simplexide, the experiments to compare natural and synthetic compound were done in polystyrene plates coated with various concentrations of glycolipids dissolved in methanol. Solvent was dried under nitrogen immediately before the addiction of cells. In another group of experiments, monocytes were preincubated with increasing concentrations of DPPE-PEG (1–30 µg/ml; 5 min) and then stimulated (37°C, 24 h) with simplexide (10 µM), α-GalCer (50 µM) or LPS (100 ng/ml). At the end of the experiment, the supernatant was removed, centrifuged (1,000 g, 4°C, 5 min) and stored at −80°C for subsequent determination of IL-6, TNF-α, IL-10, and CXCL8 release. The cells remaining in the plates were lysed with 0.1% Triton X-100 for total protein quantification by a Bradford-based assay (Biorad).

C1R and CD1d-transfected C1R were incubated (37°C, 24 h) in X-VIVO alone or with simplexide (10 µM), KRN7000 (1 µM) or PMA (100 ng/ml).

To assess iNKT expansion *in vitro*, PBMC were cultured in RPMI supplemented with 1% ultraglutamine, 1% antibiotic-antimycotic solution and 5% human AB serum and stimulated with simplexide (100 nM) or α-GalCer (100 nM). On day 2, IL-2 (100 U/ml) was added to each well. On day 7, cells were harvested and the percentage of iNKT cells among CD3^+^ cells was assessed by flow cytometry using PE-anti-TCR Vα24 and PerCP-anti-CD3 antibodies (Miltenyi Biotec).

### RT-PCR for IL6, TNFα, IL10, and CXCL8

Monocytes (5×10^6^/2 ml) were incubated (37°C, 2 h) in X-VIVO in 12-well plates. The cells were then washed and incubated (37°C, 3–12 h) in the presence or the absence of simplexide (10 µM). At the end of the incubation, total RNA from monocytes was extracted by SV total RNA isolation system (Promega, Madison, WI), treated with RNase-free DNase I and suspended in DEPC water. RNA concentration were assessed by spectrophotometry. One µg of total RNA was reverse transcribed with oligo(dT) (50 µM) and Superscript III Reverse Transcriptase (200 U, Life Technologies) as described elsewhere [30]. Real-time quantitative PCR was performed on the iCycler (Biorad) using the Platinum SYBR Green qPCR kit (Life Technologies) and target-specific primers for IL6, TNFα, IL10, CXCL8, and GAPDH as previously reported [31]. PCR efficiency and specificity were evaluated by analyzing amplification curves with serial dilutions of the template cDNA and their dissociation curves. Each cDNA sample was analyzed in triplicate and the corresponding no-RT mRNA sample was included as a negative control. The data were analyzed with iCycler iQ analysis software (Biorad), the mRNA signals in each sample were normalized to that of the GAPDH mRNA, and the changes in IL6, TNFα, IL10 and CXCL8 mRNAs were expressed as fold increase in treated vs. unstimulated cells.

### Cytokine Assay

The release of IL-6, TNF-α, IL-10 and CXCL8 in the culture supernatant was measured in duplicate determinations by commercially available ELISA kits (R&D, Minneapolis, MN USA) according to the manufacturer's instructions. Since the number of adherent monocytes can vary in each well and in different experiments, the results were normalized for the total protein content in each well, determined in the cell lysates (0.1% Triton X-100) by a Bradford based assay (Biorad).

### Silencing of CD1d

Silencing of CD1d was performed with HyPerfect Transfection Kit (Qiagen, Italy) by using four different FlexiTube siRNAs (silencing RNAs) for CD1d (Qiagen, Hs_CD1D_1, Hs_CD1D_2, Hs_CD1D_4, Hs_CD1D_5), and the validated irrelevant Allstars siRNAs (Qiagen) used as negative control. Target sequences of the FlexiTube siRNAs were: Hs_CD1D_1 (siRNA-S1)  =  CCGGTTGTGAAACCTACTGAA, Hs_CD1D_2 (siRNA-S2)  =  CAGAAGTGCAAAGGTGTGCAA, Hs_CD1D_4 (siRNA-S4)  =  TGGGCTTTACCTCCCGGTTTA, and Hs_CD1D_5 (siRNA-S5)  =  TGGGCTTTACCTCCCGGTTTA. The transfection protocol was as follows: adherent monocytes (10^6^ per sample) were incubated (37°C, 18 h) in 6-well plates with OptiMEM (Life Technologies) supplemented with 12 µl of HyPerfect Transfection Reagent (Qiagen) and 750 ng/ml of each siRNA oligonucleotides. At the end of the incubation, the transfection medium was removed, replaced with RPMI containing 10% FCS, 2 mM L-glutamine and 1% antibiotic-antimycotic solution, and the cells were incubated (37°C) for additional 72 h. At the end of the transfection protocol the amount of CD1d was assessed by western blot. To this aim, the cells were lysed in lysis buffer (20 mM Tris pH 7.5, 5 mM EDTA, 1 mM PMSF, 2 mM benzamidine, 10 µg/ml leupeptin, 10 mM NaF, 150 mM NaCl, 1% Nonidet P-40 and 5% glycerol). Equal protein extracts (25 µg per sample) were separated on 4–12% Bis-Tris gels (NuPAGE^®^, Novex, Life Technologies) and transferred to a nitrocellulose membrane (Schleicher & Schuell, Dassel, Germany). Membranes were then probed with the anti-CD1d (Clone NOR3.2/13.17), or anti-GAPDH Abs followed by HRP-conjugated secondary Abs. Membrane-bound Abs were visualized with the ECL detection system (GE Healthcare, Milan, Italy) and digitalized under the image analysis system ChemidocXRS (Biorad). Densitometric measurement of the signal intensity was performed with the Quantity One software (Biorad) and quantification of CD1d was obtained by calculating the ratio CD1d/GAPDH in each sample.

### Statistical analysis

The data are expressed as mean values ± SE of the indicated number of experiments. Statistical analysis was performed by one-way analysis of variance (ANOVA) followed by Dunnett's test (when comparison was made against a control) or Bonferroni's test (when comparison was made between each pair of groups) by means of Analyse-it for Microsoft Excel, version 2.16 (Analyse-it Software, Ltd.). A *p* value of 0.05 or lower was considered to be significant.

## Results

### Simplexide induces the release of cytokines and chemokines from human monocytes

**Figure 3:**
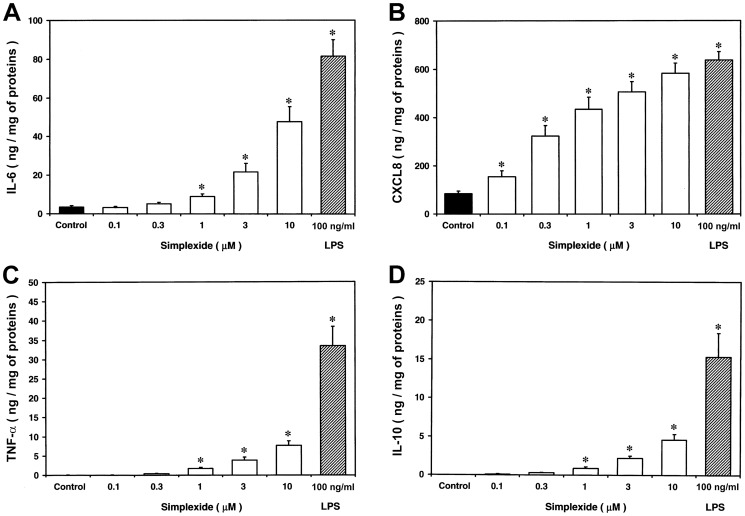
Effect of increasing concentrations of simplexide on IL-6 (panel A), CXCL8 (panel B), TNF-α (panel C) and IL-10 (panel D) release from monocytes. Monocytes were incubated (37°C, 8 h for TNF-α and IL-10 or 24 h for IL-6 and IL-8) with X-VIVO alone (control) or with the indicated concentrations of simplexide or LPS. At the end of incubation, the supernatant was collected and centrifuged (1000×*g*, 4°C, 5 min). Cytokines and chemokines were determined by ELISA. The values are expressed as ng of IL-6, CXCL8, TNF-α or IL-10 per mg of total proteins. Data are representative of ten independent experiments.

In a first group of experiments we examined the effects of simplexide on cytokine and chemokine production by human monocytes. Cells were incubated with increasing concentrations (0.1–10 µM) of simplexide and the release of cytokines and chemokines (IL-6, TNF-α, IL-10 and CXCL8) was determined. Simplexide induced a concentration-dependent release of IL-6, CXCL8 and, to a lesser extent, TNF-α and IL-10 (Fig. 3A–D). Simplexide was more potent in inducing the release of CXCL8 (EC_50_ = 0.4±0.07 µM) than IL-6 (EC_50_ = 1.2±0.02 µM), TNF-α (EC_50_ = 2.1±0.02 µM) and IL-10 (EC_50_ = 2.2±0.04 µM). The production of CXCL8 induced by simplexide was comparable to that of LPS (100 ng/ml), a well-characterized stimulus for these cells [32], whereas the release of IL-6, TNF-α and IL-10 was lower (Fig. 3A–D).

Although we used highly purified simplexide in these experiments, being of natural origin it may contain trace contaminants that could potentially activate monocytes. To exclude that the effect of simplexide could be due to LPS contamination, monocytes were stimulated with simplexide in the presence of polymyxin B (50 µg/ml), a potent inactivator of LPS [33]. Polymyxin B did not influence the capacity of simplexide to induce the release of IL-6, CXCL8 and TNF-α, whereas it almost completely suppressed the production of cytokines and chemokines induced by LPS (Table 1). To further exclude the possibility of monocyte activation due to trace contaminants other than LPS, we compared the effect of natural simplexide with that of its synthetic analogue. In these experiments, glycolipids were dissolved in methanol because synthetic simplexide was remarkably less soluble in DMSO than natural simplexide. This phenomenon can be explained by the different molecular species present in natural and synthetic simplexide. In fact, natural simplexide is a mixture of many homologous molecular species, whereas synthetic simplexide is chemically homogeneous. While the molecular heterogeneity of natural simplexide is unlikely to affect its biological properties, it could explain its higher solubility. In a recent paper [34], the chemical heterogeneity of some plant polysaccharides has been interpreted in terms of a reduced propensity to aggregation and, therefore, of increased solubility. Similarly, the heterogeneous natural simplexide is expected to be more soluble than the chemically homogeneous synthetic compounds.

**Table 1:** Effect of polymyxin B on simplexide- and LPS-induced release of IL-6, CXCL8 and TNF-α from monocytes.

	*IL-6 (ng/mg of proteins)*		*CXCL8 (ng/mg of proteins)*		*TNF-α (ng/mg of proteins)*	
	Control	+ polymyxin B	Control	+ polymyxin B	Control	+ polymyxin B
Untreated	3.5±0.7	3.4±0.7	112.9±11.6	113.±11.51	0.1±0.1	0.1±0.1
Simplexide	52.0±9.4*	54.3±9.4*	615.5±37.2*	600.9±26.9*	8.9±3.6*	9.2±4.1*
LPS	88.3±5.4*	14.9±0.3†	638.1±15.1*	29.8±0.7†	54.9±8.0*	3.5±0.9†

Monocytes were incubated for 8 h (TNF-α) or 24 h (IL-6 and CXCL8) with simplexide (10 µM) or LPS (100 ng/ml) either in the absence (Control) or the presence of polymyxin B (50 µg/ml). Data are the mean ± SE of three experiments.

* p<0.05 vs. respective untreated.

† p<0.05 vs. respective control.

**Figure 4:**
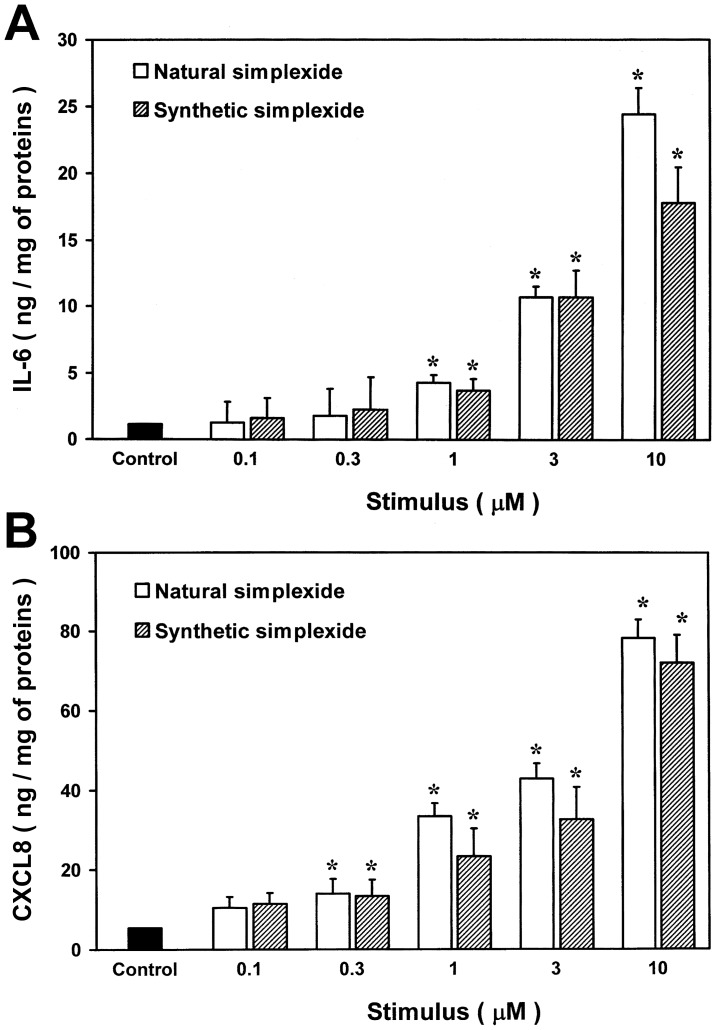
Effect of increasing concentrations of natural or synthetic simplexide on IL-6 (panel A) and CXCL8 (panel B) release from monocytes. Polystyrene plates were coated with indicated concentrations of glycolipids dissolved in methanol. Solvent was dried under nitrogen immediately before the addiction of cells. After 24 hours of incubation, the supernatant was collected and centrifuged (1000×*g*, 4°C, 5 min). Cytokines and chemokines were determined by ELISA. The values are expressed as ng of IL-6 or CXCL8 per mg of total proteins. Data are the mean ± SEM of six experiments. * p<0.05 *vs*. control.

To avoid any effect due to different solubility, we precoated the plate with the glycolipids before the addition of the cells. Figure 4 shows that natural and synthetic simplexide induced similar release of both IL-6 (A) and CXCL8 (B) from monocytes. Under these experimental conditions, the amount of cytokines produced by monocytes is lower than that reported in Figure 3, when simplexide was added directly to the cell suspension. The comparable effects of the natural and synthetic simplexide confirm that activation of monocytes is directly due to the glycolipid and not to contaminants.

**Figure 5:**
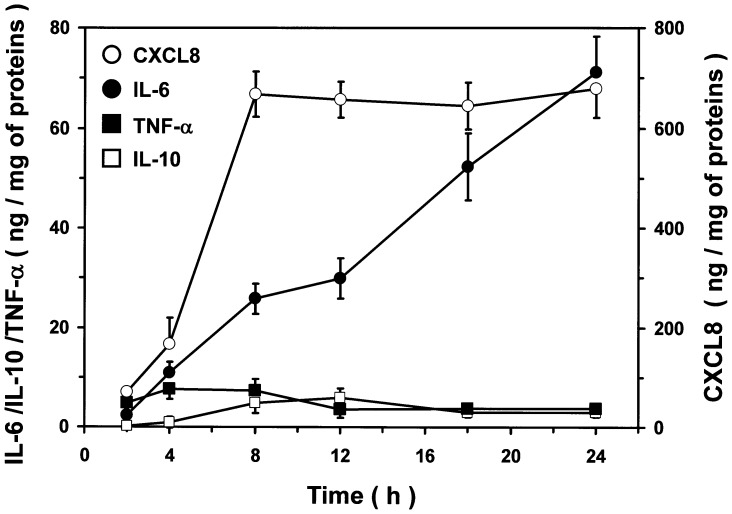
Kinetics of IL-6, CXCL8, TNF-α and IL-10 release from monocytes induced by simplexide. The cells were incubated (37°C, 3–24 h) with simplexide (10 µM). At the end of incubations, the supernatant was collected and centrifuged (1000×*g*, 4°C, 5 min). Cytokines and chemokines were determined by ELISA. The values are expressed as ng of IL-6, CXCL8, TNF-α or IL-10 per mg of total proteins. Data are the mean ± SEM of five experiments.

In the next group of experiments we examined the kinetics of IL-6, CXCL8, TNF-α and IL-10 release from monocytes stimulated with an optimal concentration of simplexide (10 µM). Figure 5 shows that production of CXCL8 began at 4 h and reached a plateau after 8 h, whereas IL-6 release progressively increased up to 24 h. Moreover, simplexide-induced release of TNF-α was rapid, reaching a maximum at 4 h and declining thereafter. Finally, the production of IL-10 was maximal between 8 and 12 h. Collectively, these results indicate that the release of cytokines/chemokines from human monocytes induced by simplexide follows distinct kinetics.

### Simplexide increases cytokine and chemokine mRNA in monocytes

**Figure 6:**
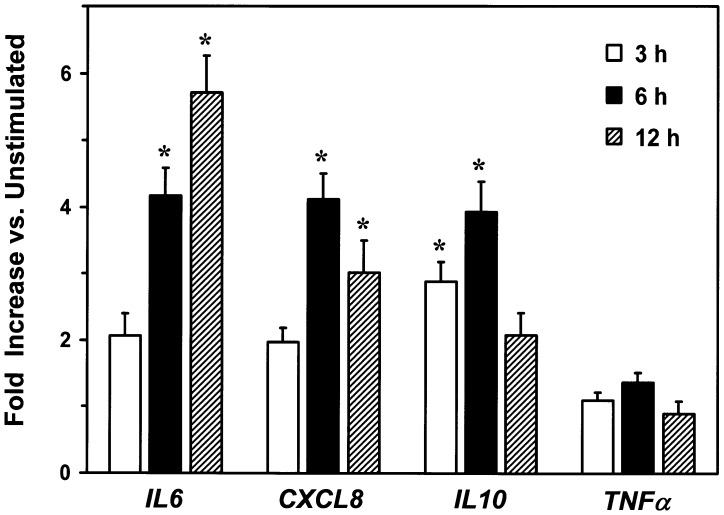
Effect of simplexide on IL-6, CXCL8, IL-10 and TNF-α mRNA expression in monocytes. The cells were incubated (37°C) for different time periods (3, 6, or 12 h) with simplexide (10 µM) or vehicle alone. mRNA levels for *IL6*, *CXCL8*, *IL10* and *TNFα* were quantitated by real-time PCR (see Methods). Expression of *IL6*, *CXCL8*, *IL10*, and *TNFα* (normalized for *GAPDH*) was expressed as fold changes *vs.* untreated cells. Data are the mean ± SE of five experiments. *p<0.05 vs. untreated.

The kinetics of cytokine/chemokine production shown in Fig. 5 suggested that simplexide may differentially modulate their mRNA expression. Thus, we examined whether simplexide activates cytokine/chemokine gene expression in monocytes by real-time quantitative PCR. Simplexide significantly enhanced mRNA expression of IL6, CXCL8, and IL10. By contrast, simplexide did not influence the expression of TNFα at any time point examined. Again, the kinetics of induction were quite different, since IL6 mRNA progressively increased up to 12 hours (5.7±0.5, fold change over unstimulated cells) whereas CXCL8 and IL10 peaked at 6 hours (4.1±0.4 and 3.9±0.5 fold, respectively) and slightly declined after 12 hours (Fig. 6). These experiments confirm that different profiles of cytokine/chemokine production induced by simplexide are paralleled by different kinetics of mRNA expression. Interestingly, simplexide did not increase TNFα mRNA expression, suggesting that the release of this cytokine is due to secretion of preformed TNF-α from intracellular stores rather than de novo gene expression.

### CD1d is required for monocyte activation induced by simplexide

**Figure 7:**
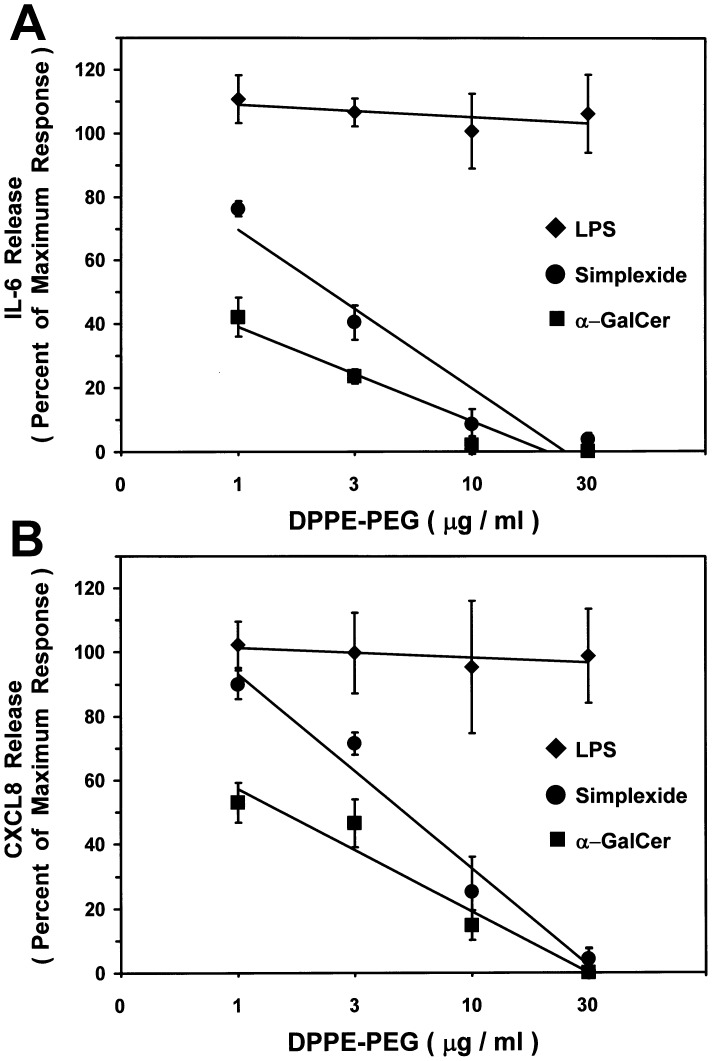
Effect of DPPE-PEG on simplexide-induced IL-6 (panel A) and CXCL8 (panel B) release from monocytes. Monocytes were preincubated (37°C, 5 min) with X-VIVO alone, or with the indicated concentrations of DPPE-PEG (1–30 µg/ml) and then stimulated (37°C, 24 h) with simplexide (•; 10 µM), α-GalCer (▪; 50 µM) or LPS (♦; 1 µg/ml). At the end of incubation, the supernatant was collected and centrifuged (1000×*g*, 4°C, 5 min). Cytokines and chemokines were determined by ELISA. The values are expressed as ng of IL-6 or CXCL8 per mg of total proteins. Data are expressed as percent inhibition of the maximum response induced by simplexide or LPS alone calculated as (R−R_b_)/(R_max_−R_b_) ×100, where R is the release in samples treated with the agonists plus DPPE-PEG, R_b_ is the release in unstimulated samples and R_max_ is the release in samples stimulated with agonists alone. Data are the mean ± SEM of five experiments. The lines represent the best fit for inhibition of simplexide, α-GalCer or LPS.

Since CD1d is the main target of glycolipids, we hypothesize that it could be involved in simplexide-induced activation of human monocytes. Thus, we used three different experimental approaches to test this hyphotesis. First, we evaluated the effect of a sterically stabilized liposome composed of dipalmitoyl-phosphatidylethanolamine covalently attached to polyethyleneglycol (DPPE-PEG). Previous studies have shown that DPPE-PEG is a potent CD1d antagonist and inhibits α-GalCer-induced activation of iNKT cells both *in vivo* and *in vitro* [35], [36]. Figure 7 (A–B) shows that DPPE-PEG concentration-dependently inhibited simplexide-induced IL-6 and CXCL8 production from monocytes with similar IC_50_ (2.8 µg/ml and 7.2 µg/ml for IL-6 and CXCL8, respectively). The same pattern of response was observed when α-GalCer was used instead of simplexide (Fig. 7A–B). The specificity of the inhibitory effect of DPPE-PEG was supported by the observation that this compound had no effect on LPS-induced IL-6 and CXCL8 production (Fig. 7A–B). The maximum response (in ng/mg of proteins) of used stimuli was for IL-6: simplexide  = 52.0±5.9; α-GalCer  = 20.2±3.7; LPS  = 81.5±8.3 *vs.* unstimulated cells 2.84±0.5; and for CXCL8: simplexide  = 625.2±37.6 ng/mg of proteins; α-GalCer  = 380.6±35.7; LPS  = 637.5±55.6 *vs.* unstimulated cells 61.8±9.8.

**Figure 8:**
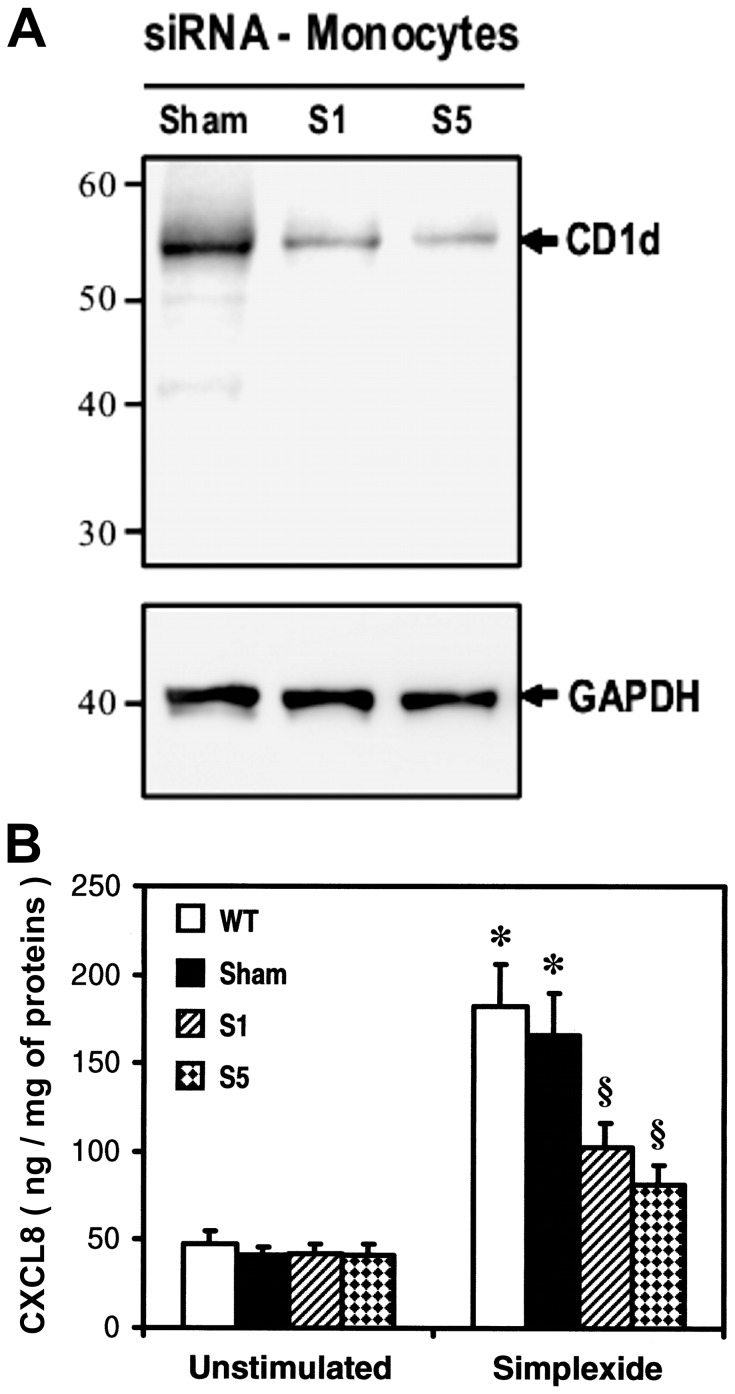
Effect of CD1d silencing on simplexide-induced release of CXCL8. Silencing of CD1d was performed as described in “Methods”. (A) Western blot of monocytes transfected with two siRNA oligonucleotides (S1 and S5) or an irrelevant oligonucleotide (Sham). The immunoblot shown is representative of three different experiments. (B) Monocytes non-transfected (WT) or transfected with siRNA oligonucleotides (S1 and S5) or Sham were incubated (37°C, 24 h) with X-VIVO alone (unstimulated) or simplexide (10 µM). At the end of incubation, the supernatant was collected and centrifuged (1000×*g*, 4°C, 5 min). CXCL8 was determined by ELISA. The values are expressed as ng of CXCL8 per mg of total proteins. Data are the mean ± SEM of four experiments. * p<0.05 *vs*. the respective unstimulated. § p<0.05 *vs*. Sham.

In a second group of experiments, we silenced CD1d expression by using different siRNA oligonucleotides. Figure 8A shows a representative experiment in which CD1d expression in transfected monocytes was evaluated by western blot. Two siRNA oligonucleotides (S1 and S5) markedly reduced CD1d protein in monocytes as compared to cells treated with irrelevant oligonucleotides (Sham). Two other siRNA oligonucleotides, S2 and S4, did not significantly reduce CD1d protein (data not shown). Densitometric analysis of three experiments showed that CD1d content was reduced by 60.5±6.5% and 67.9±7.6% with S1 and S5, respectively (p<0.05 *vs*. sham), indicating that siRNA oligonucleotides successfully knocked down CD1d. Interestingly, CD1d knock down but not sham transfection significantly reduced CXCL8 release induced by simplexide (Fig. 8B), whilst monocyte production of CXCL8 in response to LPS was unaltered (data not shown).

**Figure 9:**
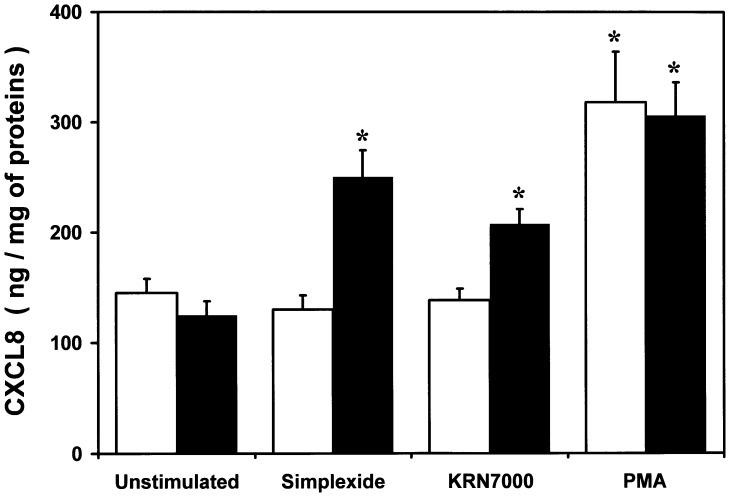
Effect of simplexide on C1R and C1R-CD1d cell lines. C1R and C1R-CD1d were incubated (37°C, 24 h) with X-VIVO alone (unstimulated), simplexide (10 µM), KRN7000 (1 µM) or PMA (100 ng/ml). At the end of incubation, the supernatant was collected and centrifuged (1000×*g*, 4°C, 5 min). CXCL8 was determined by ELISA. The values are expressed as pg of CXCL8 per mg of total proteins. Data are the mean ± SEM of six experiments. * p<0.05 *vs*. the respective unstimulated.

To unambiguously confirm that CD1d was necessary for simplexide-induced cell activation, we employed a human lymphoblastoid cell line (C1R) stably transfected with CD1d. [29] Both transfected (C1R-CD1d) and parental (C1R) cells were stimulated with simplexide (10 µM), KRN7000 (1 µM), or PMA (100 ng/ml). Figure 9 shows that simplexide induced a significant CXCL8 production by C1R-CD1d, but not C1R. Similar response was observed when C1R-CD1d and C1R were stimulated with the synthetic α-GalCer (KRN7000). By contrast PMA, which directly activates PKC [37], induced comparable release of CXCL8 in both cell lines.

### Simplexide induce the expansion of human iNKT cells in vitro

**Figure 10:**
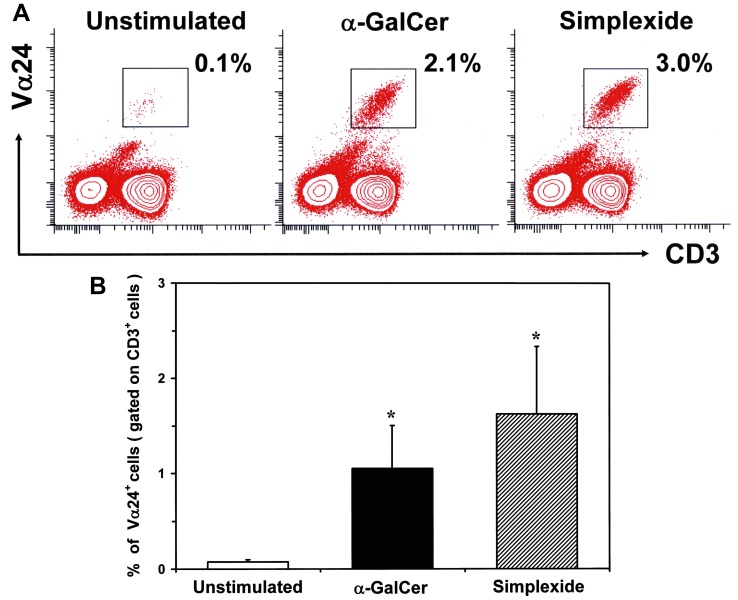
In vitro expansion of human iNKT cells. Human PBMC were stimulated with α-GalCer (100 nM) or simplexide (100 nM) for 7 days. iNKT expansion has been determined as percentage of Vα24^+^ cells among CD3^+^ lymphocytes. (A) Representative contour plots are shown. (B) Data are the mean ± SEM of four experiments. * p<0.05 *vs*. unstimulated.

As glycolipids presented by CD1d are recognized by and mediate the activation of iNKT, we asked whether simplexide could also induce iNKT cell expansion *in vitro*. To this aim, we incubated human PBMC with simplexide (100 nM) or α-GalCer (100 nM) in the presence of IL-2 (100 U/ml) [38]. After 7 days, the percentage of CD3^+^Vα24^+^ cells was assessed by flow cytometry. Figure 10A illustrates the results of a typical experiment indicating that both α-GalCer and simplexide increased the percentage of iNKT cells. The results of four experiments summarized in Fig. 10B demonstrates that both α-GalCer and simplexide significantly induced an expansion of iNKT cells.

## Discussion

In this study we demonstrate that simplexide is a glycolipid that induces expression and release of cytokines and chemokines from human monocytes. Simplexide is almost as effective in inducing IL-6 and CXCL8 production as LPS. Natural and synthetic simplexide exert comparable effects, thus indicating that the stimulatory activity is due to the glycolipid molecule rather than trace contaminants. Simplexide activates the expression of IL6, CXCL8 and IL10, but not TNFα mRNA a gene whose transcription is induced by LPS in monocytes [39]. However, a small albeit transient release of TNF-α is induced by simplexide. Interestingly, agonist-induced release of preformed TNF-α has been previously reported in immunologically activated human mast cells [40], [41]. Our data suggest that preformed TNF-α is stored also in human monocytes and that simplexide activates the release of this cytokine independently of gene transcription. The effects of simplexide on cytokine and chemokine release are mediated by CD1d expression on human monocytes. Finally, we show that simplexide expands iNKT cells in vitro to the same degree as α-GalCer.

According to the current hypothesis, stimulation of CD1d-expressing APC by glycolipids requires the concomitant presence of iNKT cells [17], [18], [19], [42]. To discriminate whether the simultaneous presence of iNKT cells is mandatory for simplexide-induced cytokine production from monocytes, or this glycolipid can itself generate intracellular signals directly by interaction with CD1d, this study was performed with highly purified preparations of monocytes, where contaminant iNKT cells were 0.0%. The profile of cytokines/chemokines induced by simplexide in monocytes suggests that this glycolipid may potentially exert both proinflammatory and immunoregulatory activities. Both CXCL8 and TNF-α are potent mediators of inflammation and are involved in recruitment and activation of inflammatory cells. On the other hand, IL-6 and IL-10 are regulatory cytokines that can at least partially explain the immunosuppressive activity previously shown by simplexide in a murine model of T cell activation [27].

The results of our study support the hypothesis that simplexide-induced activation of monocytes is dependent on CD1d expressed by these cells. First, the aglycon of simplexide is a secondary alcohol without further functional groups, glycosylated by a disaccharide chain composed of an inner α-galactose and an outer glucose that possesses α anomeric configuration, similar to that involved in the immunoregulatory activity of α-GalCer [16]. Both molecules contain two long saturated alkyl chains that can fit into the CD1d lipid-binding groove. Thus, it is reasonable to hypothesize that simplexide binds to CD1d in a similar way as α-GalCer [13]. In addition, cytokine release induced by simplexide is suppressed by a CD1d antagonist, DPPE-PEG, and by CD1d knockdown. Finally, simplexide stimulates cytokine production by CD1d-transfected C1R cells whereas it has no effect on parental C1R cell line.

Two different groups have demonstrated that CD1d crosslinking by a monoclonal anti-CD1d antibody induces the release of cytokines from human monocytes [21] and intestinal epithelial cells [22]. The latter finding has been confirmed and extended by showing that CD1d crosslinking results in increased production of IL-10 in a model of inflammatory bowel disease [23]. We have confirmed that anti-CD1d induces IL-10 production from human monocytes provided that the anti-CD1d monoclonal antibody is bivalent and is able to cross-link CD1d molecules (Loffredo *et al*, in preparation). Although simplexide and anti-CD1d elicit similar responses, it is currently unknown whether simplexide interacts with CD1d on monocytes as a monovalent or bivalent agonist. The molecular interaction between simplexide and CD1d should be further investigated.

Naturally occurring glycolipids are increasingly recognized as modulators of innate and adaptive immunity [11], [43]. Glycolipids can function as antigens primarily by interacting with CD1d expressed on APC [44]. Presentation of CD1d-bound glycolipids to NKT cells activates effector functions and cytokine production in NKT cells and subsequent transactivation of APC [18], [19], [20], [45]. This canonical model of glycolipid-induced activation of immune responses has been largely investigated using α-GalCer and related molecules. We found that simplexide, akin to α-GalCer, induces the expansion of iNKT cells *in vitro*. It will be interesting to assess the cytokine profile expressed by iNKT cells in response to simplexide and whether this differ from that induced by α-GalCer.

Taken together, our findings demonstrate that simplexide can modulate the activity of different immune cells by inducing monocyte production of cytokines and chemokines in a CD1d-dependent manner and the expansion of iNKT *in vitro*. Further studies are required to understand the potential immunological applications of simplexide. Immunologically active glycosphingolipids, α-GalCer and its synthetic analog KRN7000, are currently being evaluated in cancer immunotherapy [46], [47], [48] and as vaccine adjuvants [49]. In addition, recent findings highlight the important role of CD1d in determining the host response to environmental stimuli [23]. The observation that simplexide is a novel activator of human monocytes and iNKT cells *in vitro* raises interesting perspectives on the therapeutic potential of this glycolipid.

## Supporting Information

Figure S1 **Reagents and conditions for preparation of synthetic simplexide.** *a*. oxalyl chloride, DMSO, DCM, then Et_3_N; *b*. Mg, Et_2_O; *c*. Et_2_O; *d*. BnBr, NaH, DMF; *e*. AcOH, HCl; *f*. CCl_3_CN, Cs_2_CO_3_, DCM; *g*. PhCH(OCH_3_)_2_, TfOH, DMF; *h*. BnBr, NaH, DMF, TBAI; *i*. TES, TFA, DCM; j. TMSOTf, Et_2_O; *k*: H_2_, 20% Pd(OH)_2_/C, EtOH, AcOH; *l*: Ac_2_O, AcOH, H_2_SO_4_; *m*. NH_2_NH_2_·AcOH, DMF; *n*. CCl_3_CN, Cs_2_CO_3_, DCM; *o*. TMSOTf, DCM; *p*. Et_3_N, MeOH. Ac =  acetyl, Bn =  benzyl, DCM =  dichloromethane, DMF = *N*,*N*-dimethylformamide, DMSO =  dimethylsulfoxide, Et =  ethyl, Ph =  phenyl, TBAI =  *tert*-butylammonium iodide, TES =  trimethylsilane, Tf =  trifluoromethanesulfonate, TFA =  trifluoroacetic acid, TMS =  trimethylsilyl.
(DOC)
