# Supplementary figures and images for: Simplexide Induces CD1d-Dependent Cytokine and Chemokine Production from Human Monocytes
Source: PLoS One. 2014 Nov 12;9(11):e111326. doi: 10.1371/journal.pone.0111326 (PMC4229102; doi:10.1371/journal.pone.0111326)

**Figure S1**


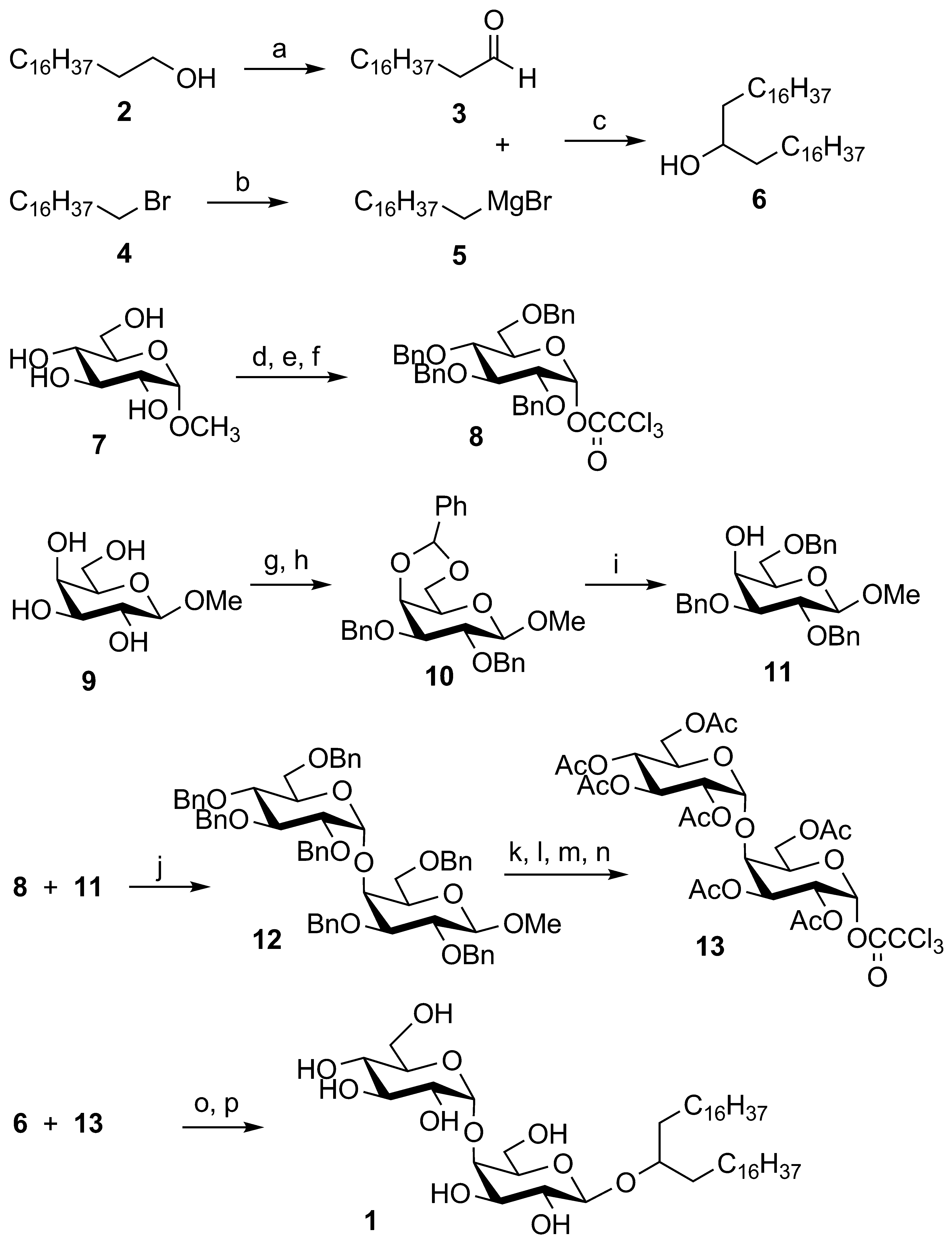

Supplement: Figure S1 — Reagents and conditions for preparation of synthetic simplexide. a. oxalyl chloride, DMSO, DCM, then Et3N; b. Mg, Et2O; c. Et2O; d. BnBr, NaH, DMF; e. AcOH, HCl; f. CCl3CN, Cs2CO3, DCM; g. PhCH(OCH3)2, TfOH, DMF; h. BnBr, NaH, DMF, TBAI; i. TES, TFA, DCM; j. TMSOTf, Et2O; k: H2, 20% Pd(OH)2/C, EtOH, AcOH; l: Ac2O, AcOH, H2SO4; m. NH2NH2·AcOH, DMF; n. CCl3CN, Cs2CO3, DCM; o. TMSOTf, DCM; p. Et3N, MeOH. Ac = acetyl, Bn = benzyl, DCM = dichloromethane, DMF = N,N-dimethylformamide, DMSO = dimethylsulfoxide, Et = ethyl, Ph = phenyl, TBAI = tert-butylammonium iodide, TES = trimethylsilane, Tf = trifluoromethanesulfonate, TFA = trifluoroacetic acid, TMS = trimethylsilyl. (DOC) [file pone.0111326.s001.doc]
